# Diagnostic value of “River Sign” in focal organizing pneumonia

**DOI:** 10.1097/MD.0000000000044894

**Published:** 2025-10-03

**Authors:** Ying Liu, Dong-ning Li, Hao Yu, Jin Huang, Lian-jun ZHou, Lin Wang, Chang-fu Wang, Xiao-ling Gao, Pei-xin Cong

**Affiliations:** a Department of CTMR, Ningcheng County Central Hospital, Chifeng, China; b Department of Gastroenterology, Ningcheng County Central Hospital, Chifeng, China.

**Keywords:** nodular or mass type, organizing pneumonia, River Sign, tomography, x-ray computed

## Abstract

Organizing pneumonia (OP) is a subset of interstitial pneumonia characterized by the clinical syndrome of organizing granulation tissue within the alveoli, alveolar ducts, or bronchioles. Histopathologically, it is marked by the presence of organizing components predominantly consisting of proliferative fibroblasts within the alveolar spaces. Focal organizing pneumonia (FOP) typically manifests as solitary pulmonary nodules or masses, constituting approximately 10% to 15% of OP cases. This condition is frequently misdiagnosed as lung cancer, which can result in unnecessary interventions such as surgical resection and percutaneous biopsy. Consequently, it is imperative to identify distinguishing features between the 2 conditions prior to surgical intervention. This study introduces a distinctive computed tomography feature, termed the “River Sign,” with the objective of enhancing radiologists’ comprehension of FOP, thereby improving diagnostic accuracy and informing clinical management strategies.

## 1. Introduction

Organizing pneumonia (OP), a specific type of interstitial pneumonia, is pathologically characterized by the presence of organizing components dominated by proliferating fibroblasts within the alveolar lumen ^[1]^. OP typically presents with patchy opacities and consolidation. As a manifestation of focal organizing pneumonia (FOP), nodular or mass-like organizing pneumonia accounts for approximately 10% to 15% of all OP cases ^[1]^, and it is easily misdiagnosed as lung cancer ^[2]^. Consequently, patients may undergo overtreatment such as surgical resection or percutaneous biopsy ^[3, 4]^. Therefore, in-depth analysis of its computed tomography (CT) findings and exploration of key imaging features for differentiation from malignant tumors are of great importance.

## 2. Materials and methods

### 2.1. General information

A retrospective analysis was conducted on 30 cases of OP, confirmed through pathological examination, from January 2019 to October 2024. The study received approval from the ethics committee (Protocol NYLLWYH2025-021). Of these, 20 cases presenting with nodular or mass-like lesions were selected as the subjects of this research. The cohort comprised 19 males and 1 female, with necrosis observed in 19 cases. The patients’ ages ranged from 39 to 80 years, with a mean age of 59.65 ± 11.09 years. The primary clinical symptoms included cough and expectoration. Additionally, 5 patients (25%) exhibited hemoptysis, and 3 patients (15%) experienced chest pain. Laboratory analyses revealed that 15 cases had elevated high-sensitivity C-reactive protein (hs-CRP) levels exceeding 5 mg/L, and 3 cases had D-dimer levels >0.5 μg/mL. Seventeen patients underwent tumor marker evaluations, which included carcinoembryonic antigen, carbohydrate antigens (CA-125, CA-199, CA-153, CA-724), cytokeratin 19 fragment, and neuron-specific enolase (NSE). Notably, 1 patient exhibited an NSE level of 20.47 ng/mL, exceeding the normal range of 0 to 16.3 ng/mL.

### 2.2. Instruments and methods

In this study, ten cases were evaluated utilizing the SIEMENS SOMATOM Force dual-source CT scanner, with scanning parameters set at a tube voltage of 120 kV and a tube current of 250 mA. Additionally, 5 cases were assessed using the SIEMENS SOMATOM 64-slice spiral CT, while another 5 cases were examined with the Philips 64-slice spiral CT. All patients were positioned supine with their arms elevated and placed alongside the head. The head-first approach was employed, and all examinations were conducted at the end of inspiration with breath-holding. The scanning parameters included a tube voltage of 120 kV, automatic tube current adjustment, and both slice thickness and interval set at 5 mm. Thin-layer reconstructions were performed at 0.75 mm for lung windows and 1 mm for mediastinal windows. For contrast-enhanced scanning, iohexol injection, with a concentration of 350 mgI/mL, manufactured by Beilu Pharmaceutical Co., Ltd., was utilized. The injection dose varied between 70 and 90 mL, administered via the median cubital vein using a high-pressure syringe at a rate of 2.5 to 3.0 mL/s. An automatic contrast agent tracking and triggering technique was applied.The trigger point was established at the origin of the descending aorta, with a threshold set at 100 Hounsfield units (HU). Arterial phase scanning commenced 5 seconds following automatic triggering, succeeded by venous phase scanning 30 seconds thereafter.

### 2.3. Observation indicators

The images were jointly analyzed and evaluated by 2 deputy chief physicians. Conventional assessments included the examination of the location, size, shape, lobulation, spiculation, internal structure, pleural relationship, mediastinal lymph node enlargement, degree of enhancement, and other lesion characteristics. Particular emphasis was placed on the presence of internal necrosis and any alterations in the “River Sign.” In this study, the “River Sign” was characterized by necrosis within the lesion resembling a meandering river, with the solid components on either side analogous to dense riverbanks. This was evidenced by the necrotic wall exhibiting a clear and distinct boundary, and the density and enhancement of the adjacent solid components on both sides being comparable.

## 3. Results

The chest CT findings of 20 patients with FOP were characterized as follows: (Table 1) The lesions varied in size from 2 to 10 cm and were all situated subpleurally. Among the 20 cases with nodular or mass-like lesions, 19 demonstrated necrotic features, and all displayed the “River Sign,” which included round, tubular, curved, and lake-shaped forms, located either centrally or eccentrically within the lesions. Enhanced CT scans revealed significant enhancement of the solid components. The CT value in plain scans was approximately 37.50 HU (ranging from 30 to 46 HU), and the CT value in enhanced scans was approximately 83.95 HU (ranging from 61 to 100 HU). There were 2 cases with an enhancement value <40 HU and 18 cases with an enhancement value >40 HU.

**Table 1:** CT findings.

CT findings	Cases(20)
Presented with solitary lesions/multiple lesions	18/2
In the right upper lobe	4/20
In the right middle lobe	3/20
In the right lower lobe	6/20
In the left upper lobe	2/20
In the left lower lobe	7/20
Lesions were round or oval-shaped/irregularly shaped	7/13
Clear boundaries/blurred boundaries	14/6
Lesions exhibited shallow lobulation/ deep lobulation	5/3
Short spicules/long spicules	2/3
The vascular convergence sign/the air bronchogram sign	5/4
The vacuole sign/cavity sign	2/2

CT = computed tomography.

## 4. Discussion

FOP is characterized by localized, nonspecific chronic inflammation of the pulmonary tissue. The clinical presentation and radiological features of FOP are notably nonspecific. Predominant clinical symptoms include cough, sputum production, hemoptysis, and thoracic pain. FOP is predominantly observed in middle-aged and elderly individuals, particularly those between 50 and 60 years of age. It has been documented^[5]^ that the incidence is higher in males compared to females, which aligns with the present case series where only one case involved a female patient. The CT manifestations of FOP can be categorized into nodular, mass-like, and infiltrative consolidation types.^[5]^ Due to the overlap in clinical presentation, physical examination findings, and CT characteristics, FOP is frequently mistaken for lung cancer, leading to potential misdiagnosis.^[6]^ Prior research^[2,6–10]^ has primarily concentrated on distinguishing between these conditions by evaluating features such as lesion location, shape, spiculation, and lobulation, with limited focus on the morphology of internal necrosis. This study introduces the “River Sign” concept to advance the understanding of FOP and expand the repertoire of CT indicators associated with the condition.

**Figure 1.**
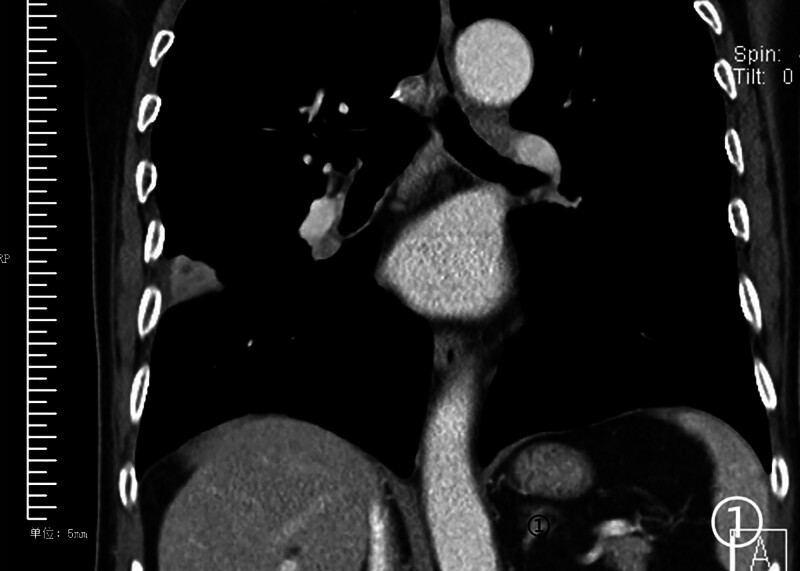
A 20-day history of intermittent right chest pain without obvious cause. Imaging revealed a solitary lesion in the posterior segment of the right upper lobe adjacent to the interlobar fissure and subpleural area, containing a round necrotic core with marked homogeneous enhancement of the surrounding solid component.

**Figure 2.**
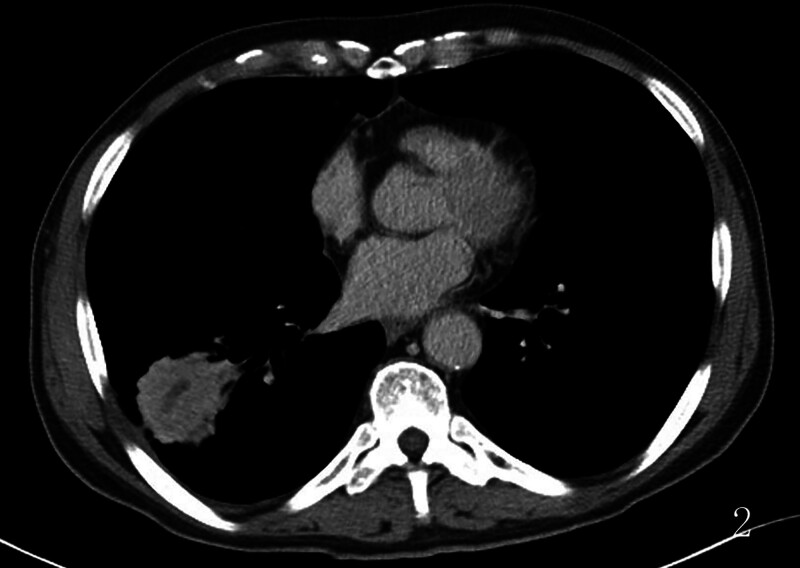
A 10-day history of paroxysmal cough with bloody sputum. Lung window imaging showed a rounded, spiculated lesion, while mediastinal window imaging revealed tubular necrosis with distinct margins and homogeneous enhancement of the surrounding parenchyma.

**Figure 3.**
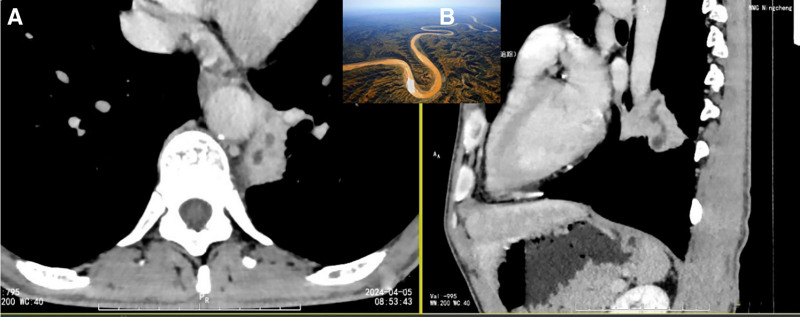
(A) Solitary peripheral lesions in the left lung with curvilinear necrosis spanning multiple planes. (B) Curved planar reconstruction demonstrated a “meandering river” morphology (such as web picture).

**Figure 4.**
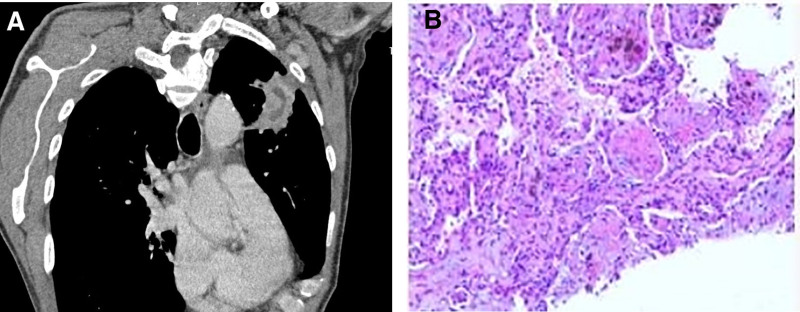
(A) Peripheral lesion in the left upper lobe of the lung; curved reconstruction shows a “meandering river” morphology. (B) Pathological examination confirmed characteristic Masson bodies.

**Figure 5.**
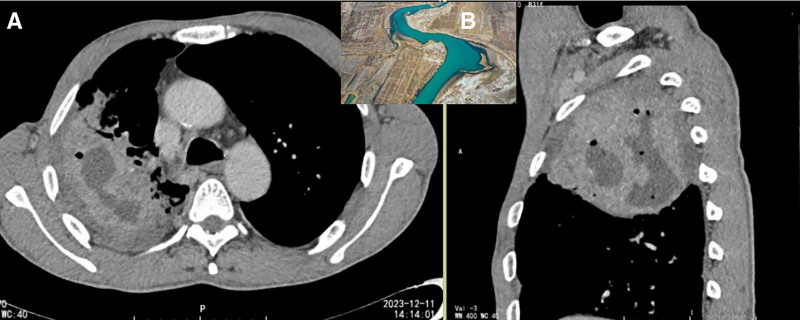
Mass-like lesions in the right upper lobe exhibited multiple round or lake-like necrotic regions (A) with well-defined borders and homogeneous enhancement of the solid components (such as web picture) (B).

In this study, the concept of the “River Sign” was introduced, defined as the presence of necrosis within nodular or mass-like lesions, characterized by a tortuous pattern reminiscent of flowing river water. The boundary of the necrotic wall is distinct, and the enhancement of the surrounding wall is pronounced, with comparable enhancement values. In this cohort, 95% of nodular or mass-like FOP cases exhibited necrosis, indicating a relatively high prevalence. This finding aligns with the observations of Liu Lantao et al,^[10]^ who reported that small FOP lesions can also demonstrate liquefaction and necrosis. Consequently, the “River Sign” can be considered a specific indicator. The necrosis may manifest as round formations, akin to small puddles (as depicted in Fig. 1), particularly when the necrosis within the lesion is minor, which is relatively common in nodular FOP. Alternatively, it may appear as columnar or tubular structures (as illustrated in Fig. 2), curved tubular forms (as shown in Figs. 3 and 4), or lake-shaped configurations (as depicted in Fig. 5). The necrosis may not reside within the same plane, analogous to a river with a winding course or forming a localized lake.

Necrosis within FOP primarily results from exudate accumulation in the alveoli or the formation of microabscesses.^[11]^ The majority of the lesion walls are attributed to the proliferation of granulation tissue, which accounts for the pronounced enhancement frequently observed in contrast-enhanced imaging.^[6]^ In this study cohort, 90% of the solid components exhibited enhancement values exceeding 40 HU, consistent with existing literature.^[9]^ The presence of “vacuoles” or “cavities” within the lesions can be attributed to the incomplete absorption of inflammatory components in the central region of the lesions. This incomplete absorption leads to encapsulation by adjacent granulation tissue and fibrous proliferation. Over time, the liquefied and necrotic materials are expelled from the body,^[12]^ resulting in smooth inner walls devoid of mural nodules.

Laboratory analysis reveals that D-dimer, a fibrin degradation product, is often elevated in conditions of hypercoagulability and hyperfibrinolysis within the human body. In patients with cancer, abnormal blood coagulation can lead to increased D-dimer levels. According to literature, the prevalence of elevated D-dimer in lung cancer patients is approximately 61.2%.^[13]^ In contrast, the prevalence in this cohort of patients with FOP was 15%, indicating a relatively low occurrence. Tumor markers such as carcinoembryonic antigen, squamous cell carcinoma antigen, CA-125, CA-199, CA-153, CA-724, NSE, and cytokeratin fragment 21-1 are frequently utilized in the assessment of lung cancer. Although these markers individually exhibit relatively low specificity, their combined use can achieve a diagnostic sensitivity of approximately 80% for lung cancer.^[14]^ In this FOP patient group, only one individual exhibited elevated NSE levels, suggesting that the assessment of D-dimer and tumor markers in FOP patients presenting with nodular or mass-like manifestations on CT scans holds diagnostic and differential diagnostic value.

### 4.1. Diagnosis and differential diagnosis

The primary differential diagnosis to consider is peripheral lung cancer. Typically, a lesion exhibiting heterogeneous density, along with characteristics such as lobulation, spiculation, vacuole sign, and spinous process, is indicative of malignancy. However, these features often overlap between the 2 conditions. In terms of internal characteristics, significant differences arise between FOP and lung cancer when liquefaction or necrosis is present in the lesion. FOP frequently exhibits a “river-like sign,” where the necrotic region assumes a relatively regular, river-like shape. Conversely, necrosis in lung cancer lesions tends to be diffuse, with poorly defined boundaries, and the extent of liquefaction or necrosis is generally larger. During contrast-enhanced scanning, the enhancement patterns of the 2 conditions also differ. The solid component of FOP typically demonstrates significant enhancement, with an increase in CT value usually exceeding 40 HU, whereas lung cancer generally exhibits mild to moderate enhancement. A thorough analysis of these imaging features aids in improving diagnostic accuracy. A detailed analysis of these imaging features helps to improve the accuracy of differential diagnosis between FOP and peripheral lung cancer.

### 4.2. This study is subject to several limitations

Firstly, the relatively small sample size may result in a biased interpretation of clinical data and CT imaging features. Secondly, for cases obtained via percutaneous biopsy, there are constraints in comprehending the “River Sign” at the pathological level. To address these issues, it is essential to increase the sample size in future studies for more comprehensive analysis and synthesis.

### 4.3. In conclusion

the presence of round, tubular, curved tubular, or lake-shaped changes resembling flowing river water with distinct boundaries within lesions, coupled with pronounced and uniform enhancement of the surrounding solid areas, can provide reliable imaging evidence for the diagnosis and differential diagnosis of FOP. This should be considered alongside the location and morphology of the lesions as well as laboratory examinations.

## Author contributions

**Conceptualization:** Ying Liu, Dong-ning Li, Jin Huang, Lian-jun ZHou.

**Data curation:** Ying Liu, Jin Huang.

**Formal analysis:** Ying Liu, Pei-xin Cong.

**Funding acquisition:** Dong-ning Li, Hao Yu, Chang-fu Wang, Pei-xin Cong.

**Investigation:** Ying Liu, Dong-ning Li, Chang-fu Wang, Xiao-ling Gao.

**Methodology:** Ying Liu, Dong-ning Li, Jin Huang, Lin Wang, Xiao-ling Gao.

**Project administration:** Hao Yu, Jin Huang, Lin Wang, Pei-xin Cong.

**Resources:** Dong-ning Li, Hao Yu, Lin Wang, Chang-fu Wang, Xiao-ling Gao.

**Software:** Hao Yu, Lian-jun ZHou, Lin Wang, Chang-fu Wang, Pei-xin Cong.

**Supervision:** Hao Yu, Lian-jun ZHou, Chang-fu Wang.

**Validation:** Ying Liu, Lian-jun ZHou, Xiao-ling Gao, Pei-xin Cong.

**Visualization:** Ying Liu, Lian-jun ZHou, Xiao-ling Gao.

**Writing – original draft:** Ying Liu.

**Writing – review & editing:** Ying Liu.
